# Supplementary material for: Dosimetry study of 3D-printed noncoplanar template-assisted CT-guided 125I seed implantation for the treatment of recurrent and metastatic tumors in the head and neck
Source: PLoS One. 2025 Aug 14;20(8):e0330271. doi: 10.1371/journal.pone.0330271 (PMC12352675; doi:10.1371/journal.pone.0330271)
Supplement: Attachment — Submitted filename: Review the article pdf .pdf [file pone.0330271.s003.pdf]

Review the article

Abstract

1- The abstract would benefit from a brief background sentence to set the context and explain why evaluating 3D-printed template-assisted implantation is clinically relevant in head and neck cancers.
2- The conclusion could be slightly expanded to comment on clinical significance or suggest the need for further validation in larger or prospective studies.

Introduction

Overall, the introduction does a good job of providing strong background context, clearly outlining the burden of head and neck cancers, the challenges in treating them, and the advantages of using ^{125}I seed implantation. It effectively explains the rationale for using 3D-printed noncoplanar templates (3DPNCTs) and references prior work and technological improvements that have contributed to this field. However, there are several areas where the introduction could be strengthened to improve clarity and scientific rigor. Below are some points to consider:

1- I think the introduction does a good job showing the benefits of 3D-printed noncoplanar templates and backing that up with previous studies. But it would be helpful if the authors briefly compared this technique with other newer approaches, like robotic-assisted implantation or adaptive radiotherapy. That would make it clearer why 3DPNCTs were chosen and what makes them stand out in this context.
2-

"Despite these advancements, several limitations remain. First, most available studies focus on verifying dosimetric feasibility and lack large-scale comparisons of preoperative and postoperative dose consistency as well as long-term efficacy evaluations[16–18]. Second, research on biological dose optimization for tumor heterogeneity (e.g., hypoxic regions or necrotic cores) remains in the exploratory phase, and the application of the linear–quadratic (L–Q) model-based equivalent dose formula (EQD2) in seed implantation has not yet been standardized[19–21]. Additionally, the cost and requirement for multidisciplinary collaboration limit the clinical adoption of 3DPNCTs, particularly in resource-limited healthcare settings"

That paragraph lists several important limitations in the current literature:

- Lack of large-scale comparisons between pre-op and post-op dosimetry
- Limited research on biological dose optimization for heterogeneous tumors
- No standardized use of EQD2 formulas
- Practical limitations like cost and need for collaboration

These are valid and well-described gaps in the field.
But the issue is: the authors don't clearly say how their own study will address those specific gaps.

For example, they could have followed that paragraph with something like: (To address these issues, this study aims to evaluate dosimetric consistency between pre- and postoperative plans and assess clinical feasibility in a resource-limited setting)

3- The study lacks a clear mention of the null and alternative hypotheses, which are essential for understanding the specific questions the authors aimed to address. Additionally, the introduction provides valuable background, but it would be helpful to explicitly state the study's aim and the expected outcomes. Could the authors clarify the hypotheses and the specific outcomes they intend to measure to strengthen the study's scientific framework?.

Patients and methods

"The clinical data of 26 patients with recurrent or metastatic head and neck tumors who were treated at Zhejiang Provincial People's Hospital between January 2018 and December 2022 were retrospectively analyzed".

1- **How was the sample size of 26 patients determined?** Did the authors perform a power analysis before the study or sample size justification? If so, which power and effect size did they use to calculate the sample size?
2- **What were the inclusion and exclusion criteria for selecting the patients in this study?** Clear criteria help in understanding the population studied and the relevance of the findings.
3- **What method was used for retrospective patient selection?** Were the patients selected based on specific clinical characteristics, diagnostic criteria, or treatment history?
4- **In the methodology section, you mention that the study includes 26 patients**, but the gender and pathological type breakdown adds up to 27 patients. The details are as follows:

- Gender: 23 male patients, 4 female patients (Total: 23 + 4 = 27 patients)

Pathological types of head and neck cancers:

Nasopharyngeal carcinoma: 14 patients

Laryngeal carcinoma: 4 patients

Hypopharyngeal carcinoma: 2 patients

Maxillary sinus carcinoma: 2 patients

Thyroid carcinoma: 2 patients

Buccal mucosal carcinoma: 1 patient

Floor of mouth carcinoma: 1 patient

Parotid gland carcinoma: 1 patient
(Total: 14 + 4 + 2 + 2 + 2 + 1 + 1 + 1 = 27 patients)

Please clarify the discrepancy between the total number of patients (26) mentioned earlier in the text and the breakdown provided here, which adds up to 27. Adjusting for consistency throughout the methodology section is recommended.

5- I noticed that the study includes patients with a variety of tumor types, which may differ in size, location, blood supply, and tissue depth. I wonder if there was any consideration to standardizing the patient group by tumor type or location to better assess the effectiveness of the 3D-printed noncoplanar template-assisted implantation technique in specific areas. It would be interesting to see if the dosimetric outcomes varied across different tumor characteristics, or if this variation could influence the overall findings.

6- The study design is retrospective, which is common in dosimetric research. However, this approach comes with some limitations. One main issue is that, since the study looks at past data, the researchers are unable to control for all factors that could affect the results (such as patient characteristics or variations in how the procedures were performed).
It would be helpful if the authors could clarify how they minimized potential biases in this study. For example, did they ensure that all patients followed standardized procedures? Were the same operators consistently performing the procedures? Addressing these points would strengthen the reliability of the results and help mitigate any potential biases.

7- While the authors state that each step of the implantation workflow is detailed, the process of designing the 3D-printed noncoplanar template remains unclear in some technical aspects.
- How the STL file was created (from what input - e.g., segmentation, external surface scanning, etc.)
- How the spatial registration between the CT scan (DICOM) and the 3D template (STL) was performed.
- How the alignment between virtual planning and printed guide was verified before use

- What software was used for merging or verifying this integration

8- A reader or another institution cannot easily reproduce the procedure without:

- Knowing the BTPS software used
- Understanding how trajectory planning was translated to physical needle guide paths

9- Steps 6 and 7 seem to describe very similar things — both talk about using a CT scan to check if the needles are in the right place and making sure the error is less than 2 mm. It's a bit hard to tell if these are two different steps or just two ways of explaining the same thing. It might be clearer if the authors either combine them or explain more clearly how they're different

"6- Preoperative Repositioning and Calibration: The patient was repositioned and then secured. A reference needle was inserted, and a validation CT scan (5 mm slice thickness) was performed. If the target registration error was ≤ 2.0 mm, implantation proceeds; if the target registration error was > 2.0 mm, a six-degree-of-freedom model was used for recalibration.

7. Verification of Needle Trajectories: The CT scan ensured proper needle placement, with corrections made until errors were ≤ 2 mm."

10- "The instruments were sterilized with wet heat."

The statement that instruments were sterilized using 'wet heat' is somewhat vague. It would be helpful to specify the exact sterilization method - for example, whether steam autoclaving was used, and if so, under what parameters (e.g., temperature and duration). Providing these details would improve clarity and reproducibility of the protocol.

11- Preoperative Treatment Planning: The BTPS was used to design the noncoplanar implantation trajectory on the basis of tumor anatomy. The seed activity (0.6–0.8 mCi) and prescribed dose (PD) were set, ensuring a D90 ≥ 95% PD for the GTV and OAR doses within acceptable limits

The dose constraints for OARs are said to be "within acceptable limits" but it would be more rigorous to clearly state whether this follows an established guideline.

Discussion

1- The discussion includes a detailed explanation of the limitations of freehand puncture techniques in head and neck brachytherapy, which is important background. However, this information was already covered in the introduction. To avoid redundancy, the authors may consider summarizing this section more briefly or rephrasing it to focus specifically on how their findings support or address these previously mentioned challenges.
2- The brief note on seed migration is appreciated, but it may be helpful to elaborate on follow-up duration and whether the study design allowed for delayed migration to be captured. A comparison with seed migration rates in the literature would also provide useful context.

3- The authors mention that integration of rapid prototyping could improve future workflows, which is promising. Expanding slightly on how automation or software advancements (e.g., intraoperative re-planning or AI-based registration) might reduce remaining sources of error would enhance the forward-looking part of the discussion.
4- The discussion would benefit from referencing previous studies to support or contrast the current findings. Including citations of studies that have shown similar results would help validate the conclusions, while mentioning studies with different outcomes could offer a deeper understanding of the variables that influence implantation accuracy and dose distribution.
5- Additionally, the manuscript would be strengthened by a more forward-looking perspective. The current mention of 'rapid prototyping' is a good start, but future research directions could include exploring real-time image-guided implantation, intraoperative adaptive planning, automated needle path planning, or AI-assisted registration systems. These advancements could further minimize deviations and improve accuracy in complex anatomical regions like the head and neck.

Conclusions

1- The conclusion restates key findings effectively, but it could be more concise by avoiding repetition of earlier points already addressed in the discussion. And also would benefit from briefly acknowledging limitations, such as the small sample size or anatomical shifts that could influence template alignment. Including these would give a more balanced and realistic summary.
2- The authors may also consider adding a sentence about future research directions, such as integrating intraoperative imaging, real-time adaptive planning, or AI-guided needle placement to further reduce intraoperative deviations and improve outcomes
