# Supplementary material for: Dosimetry study of 3D-printed noncoplanar template-assisted CT-guided 125I seed implantation for the treatment of recurrent and metastatic tumors in the head and neck
Source: PLoS One. 2025 Aug 14;20(8):e0330271. doi: 10.1371/journal.pone.0330271 (PMC12352675; doi:10.1371/journal.pone.0330271)
Supplement: Attachment — Submitted filename: Responses_to_reviewers_PLOSONE.pdf [file pone.0330271.s005.pdf]

Responses_to_reviewers_PLOSONE

Reviewer 1

Abstract

Comment 1.

The abstract would benefit from a brief background sentence to set the context and explain why evaluating 3D-printed template-assisted implantation is clinically relevant in head and neck cancers.

Response 1:

Thank you for your insightful suggestion. We agree that providing context regarding the clinical relevance of 3D-printed template-assisted implantation in head and neck cancers will improve the clarity and significance of the abstract. To address this, we have added a concise background sentence at the beginning of the abstract to better contextualize the study's rationale（**Line 21-23**）**:**

Recurrent and metastatic tumors of the head and neck often pose significant treatment challenges due to their proximity to critical structures and prior radiation exposure.

Comment 2.

The conclusion could be slightly expanded to comment on clinical significance or suggest the need for further validation in larger or prospective studies.

Response 2:

Thank you for this valuable suggestion. We agree that highlighting the clinical relevance and acknowledging the need for further validation would strengthen the impact of the abstract. Accordingly, we have revised the conclusion sentence of the abstract to reflect both the potential clinical value of 3D-printed template-guided implantation and the necessity for future large-scale or prospective studies（**Line 36-39**）**:**

These results suggest that this technique has promising clinical applicability in complex head and neck cases; however, further validation through larger prospective studies is warranted

to confirm long-term efficacy and safety.

Introduction

Comment 1.

I think the introduction does a good job showing the benefits of 3D-printed noncoplanar templates and backing that up with previous studies. But it would be helpful if the authors briefly compared this technique with other newer approaches, like robotic-assisted implantation or adaptive radiotherapy. That would make it clearer why 3DPNCTs were chosen and what makes them stand out in this context.

Response 1:

We appreciate the reviewer's thoughtful suggestion. To enhance the scientific rigor and contextual clarity of the introduction, we have added a brief comparative discussion of 3DPNCTs with emerging techniques such as robotic-assisted implantation and adaptive radiotherapy. This addition underscores the practical advantages of 3DPNCTs, particularly in terms of accessibility, anatomical adaptability, and procedural simplicity in resource-limited clinical settings（**Line 85-94**）**:**

While emerging technologies such as robotic-assisted implantation and adaptive radiotherapy offer enhanced precision and real-time adaptation to anatomical variations [16,17], they often require advanced infrastructure, extended procedural time, and specialized operator training. In contrast, 3D-printed noncoplanar templates offer a relatively low-cost, customizable solution that integrates seamlessly with conventional CT-guided workflows. Their adaptability to complex anatomical geometries and capacity for individualized needle trajectory planning make them particularly suitable for salvage treatments in the head and neck region. These practical advantages support the selection of 3DPNCTs in this study over more technologically intensive alternatives.

Comment 2.

“Despite these advancements, several limitations remain. First, most available studies focus on verifying dosimetric feasibility and lack large-scale comparisons of preoperative and postoperative dose consistency as well as long-term efficacy evaluations[16–18]. Second, research on biological dose optimization for tumor heterogeneity (e.g., hypoxic regions or necrotic cores) remains in the exploratory phase, and the application of the linear–quadratic (L–Q) model-based equivalent dose formula (EQD2) in seed implantation has not yet been standardized[19–21]. Additionally, the cost and requirement for multidisciplinary collaboration limit the clinical adoption of 3DPNCTs, particularly in resource-limited healthcare settings” That paragraph lists several important limitations in the current literature: • Lack of large-scale comparisons between pre-op and post-op dosimetry • Limited research on biological dose optimization for heterogeneous tumors • No standardized use of EQD2 formulas • Practical limitations like cost and need for collaboration These are valid and well-described gaps in the field.But the issue is: the authors don’t clearly say how their own study will address those specific gaps. For example, they could have followed that paragraph with something like: (To address these issues, this study aims to evaluate dosimetric consistency between pre- and postoperative plans and assess clinical feasibility in a resource-limited setting)

Response 2:

We thank the reviewer for this insightful observation. To improve the logical flow and clarify the objective of our study in the context of current research gaps, we have revised the end of the Introduction section. The revised version explicitly links our study goals to the limitations outlined in the previous paragraph, emphasizing how our work addresses them through a comparative dosimetric analysis and feasibility validation in a real-world, resource-constrained clinical setting（**Line 105-110**）**:**

To address these limitations, this study aims to systematically evaluate the consistency between preoperative and postoperative dosimetric parameters in 3DPNCT-assisted ^{125}I *seed implantation for head and neck tumors. Furthermore, it assesses the clinical feasibility and*

precision of this approach within a routine, resource-constrained radiotherapy environment, thereby providing evidence to support its broader clinical adoption.

Comment 3.

"The study lacks a clear mention of the null and alternative hypotheses, which are essential for understanding the specific questions the authors aimed to address. Additionally, the introduction provides valuable background, but it would be helpful to explicitly state the study's aim and the expected outcomes. Could the authors clarify the hypotheses and the specific outcomes they intend to measure to strengthen the study's scientific framework?"

Response 3:

Thank you for this insightful and constructive comment. We agree that explicitly stating the hypotheses and the study aims will improve the scientific clarity and rigor of the manuscript. In response, we have revised the final part of the Introduction to include a clear statement of the study's objectives, the null and alternative hypotheses, and the dosimetric outcomes used to evaluate the feasibility and consistency of the 3DPNCT-assisted implantation technique **（Line 111-121）：**

We hypothesized that there would be no significant differences between the preoperative and postoperative dosimetric parameters (null hypothesis, H_0), indicating accurate execution of the implantation plan. Conversely, the alternative hypothesis (H_1) posits that significant discrepancies exist, reflecting procedural variability or anatomical constraints.

The specific dosimetric outcomes evaluated include D90, D100, V100, V150, V200, CI (conformity index), HI (homogeneity index), and EI (external index), alongside the total number of seeds implanted. Bland–Altman analysis is further used to assess the consistency of each parameter. These metrics provide a comprehensive evaluation of spatial dose accuracy, plan reproducibility, and clinical feasibility.

Patients and methods

Comment 1.

"How was the sample size of 26 patients determined? Did the authors perform a power analysis before the study or sample size justification? If so, which power and effect size did they use to calculate the sample size?"

Response 1:

Thank you for this important question. We acknowledge that a formal a priori power analysis was not performed prior to data collection. The sample size of 26 patients reflects the total number of eligible cases meeting the inclusion criteria (i.e., recurrent or metastatic head and neck tumors treated with 3DPNCT-assisted ^{125}I seed implantation) over a five-year period at our institution.

This study was designed as a retrospective feasibility and dosimetric consistency assessment, not to test a specific effect size for clinical outcome endpoints. While we recognize that the limited sample size restricts the statistical power to detect small differences, the observed consistency in key dosimetric parameters provides preliminary but meaningful evidence supporting the accuracy of 3DPNCT-assisted implantation in clinical practice.

We have now clarified this point in the "Patients and Methods" section **（Line 138-142）：**

This retrospective study included all patients who met the eligibility criteria within the five-year recruitment window. No a priori power calculation was performed, as the study was exploratory in nature and focused on assessing dosimetric reproducibility rather than hypothesis-driven clinical outcome comparison.

Comment 2.

What were the inclusion and exclusion criteria for selecting the patients in this study? Clear criteria help in understanding the population studied and the relevance of the findings.

Response 2:

Thank you for pointing out this important omission. We agree that clearly defined inclusion and exclusion criteria are essential for interpreting the study results. To address this, we have

now added a dedicated description of the inclusion and exclusion criteria to the “Patients and Methods” section of the manuscript（**Line 142-151**）**:**

Patients were eligible for inclusion if they met the following criteria: (1) age ≥18 years; (2) histologically confirmed recurrent or metastatic head and neck malignancy; (3) prior external beam radiotherapy (EBRT) was completed at least 6 months before seed implantation; and (4) availability of complete preoperative and postoperative CT imaging for dosimetric evaluation. Exclusion criteria included: (1) active bleeding or uncorrectable coagulopathy; (2) KPS <60; (3) presence of distant metastases outside the head and neck region requiring systemic treatment priority; (4) incomplete dosimetric data or poor-quality imaging preventing reliable analysis.

Comment 3.

What method was used for retrospective patient selection? Were the patients selected based on specific clinical characteristics, diagnostic criteria, or treatment history?

Response 3:

Thank you for raising this important point. To improve the transparency of our methodology and reduce the risk of selection bias, we have now clarified the retrospective patient selection process. All patients were consecutively identified through the hospital’s radiation oncology treatment database using predefined diagnostic and procedural codes. Selection was based on specific clinical and treatment criteria, as detailed below（**Line 135-138,142-151**）**:**

The clinical data of 26 patients with recurrent or metastatic head and neck tumors who were treated at Zhejiang Provincial People's Hospital between January 2018 and December 2022 were retrospectively analyzed. All patients underwent 3D-printed noncoplanar template-assisted CT-guided ^{125}I seed implantation.

Patients were eligible for inclusion if they met the following criteria: (1) age ≥18 years; (2) histologically confirmed recurrent or metastatic head and neck malignancy; (3) prior external beam radiotherapy (EBRT) was completed at least 6 months before seed implantation; and (4)

availability of complete preoperative and postoperative CT imaging for dosimetric evaluation. Exclusion criteria included: (1) active bleeding or uncorrectable coagulopathy; (2) KPS <60; (3) presence of distant metastases outside the head and neck region requiring systemic treatment priority; (4) incomplete dosimetric data or poor-quality imaging preventing reliable analysis.

Comment 4.

In the methodology section, you mention that the study includes 26 patients, but the gender and pathological type breakdown adds up to 27 patients. The details are as follows:• Gender: 23 male patients, 4 female patients (Total: 23 + 4 = 27 patients)Pathological types of head and neck cancers:Nasopharyngeal carcinoma: 14 patients，Laryngeal carcinoma: 4 patients，Hypopharyngeal carcinoma: 2 patients，Maxillary sinus carcinoma: 2 patients，Thyroid carcinoma: 2 patients，Buccal mucosal carcinoma: 1 patient，Floor of mouth carcinoma: 1 patient，Parotid gland carcinoma: 1 patient(Total: 14 + 4 + 2 + 2 + 2 + 1 + 1 + 1 = 27 patients)Please clarify the discrepancy between the total number of patients (26) mentioned earlier in the text and the breakdown provided here, which adds up to 27. Adjusting for consistency throughout the methodology section is recommended.

Response 4:

We sincerely thank the reviewer for identifying this inconsistency. Upon careful re-examination of the patient database, we confirm that the total number of patients included in the study is indeed 26. The inconsistency arose due to two minor tabulation errors in the manuscript:

1.Gender breakdown: The number of male patients was mistakenly listed as 23, when it should be 22. The number of female patients is correct at 4, yielding a total of 26 patients.

2.Pathological type breakdown: The number of patients with nasopharyngeal carcinoma was incorrectly reported as 14, whereas the correct number is 13. All other cancer types and their corresponding counts were verified to be accurate（**Line 152-157）:**

Twenty-two patients were male, and 4 patients were female. The pathological types included nasopharyngeal carcinoma (13 patients), laryngeal carcinoma (4 patients), hypopharyngeal carcinoma (2 patients), maxillary sinus carcinoma (2 patients), thyroid carcinoma (2 patients), buccal mucosal carcinoma, floor of mouth carcinoma, and parotid gland carcinoma (1 patient each).

Comment 5.

I noticed that the study includes patients with a variety of tumor types, which may differ in size, location, blood supply, and tissue depth. I wonder if there was any consideration to standardizing the patient group by tumor type or location to better assess the effectiveness of the 3D-printed noncoplanar template-assisted implantation technique in specific areas. It would be interesting to see if the dosimetric outcomes varied across different tumor characteristics, or if this variation could influence the overall findings.

Response 5:

We thank the reviewer for this insightful comment. Indeed, we acknowledge that anatomical and biological heterogeneity—such as tumor depth, proximity to critical structures, and tissue rigidity—may affect needle trajectory and dosimetric reproducibility in 3DPNCT-assisted implantation. Our current study cohort includes a diverse range of recurrent or metastatic head and neck tumors, which reflects real-world clinical practice but introduces potential variability in dosimetric outcomes.

In this retrospective feasibility study, we did not stratify patients by tumor type or location, primarily due to the limited sample size. However, we agree that a subgroup analysis (e.g., deep vs. superficial tumors, or skull base vs. oral cavity lesions) would offer valuable insights into anatomical suitability and dose delivery accuracy of the 3DPNCT technique. We have now acknowledged this as a limitation in the Discussion section and outlined it as a direction for future research（**Line 421-428**）**:**

An important limitation of this study is the inclusion of patients with diverse tumor types and

anatomical locations. Variations in tumor depth, local tissue compliance, and adjacency to organs at risk may influence needle placement accuracy and, consequently, dosimetric consistency. While this heterogeneity mirrors routine clinical practice, it may obscure location-specific performance differences of the 3D-printed template system. Future studies with larger cohorts should consider stratified or subgroup analyses to evaluate anatomical factors affecting implantation precision.

Comment 6.

The study design is retrospective, which is common in dosimetric research. However, this approach comes with some limitations. One main issue is that, since the study looks at past data, the researchers are unable to control for all factors that could affect the results (such as patient characteristics or variations in how the procedures were performed). It would be helpful if the authors could clarify how they minimized potential biases in this study. For example, did they ensure that all patients followed standardized procedures? Were the same operators consistently performing the procedures? Addressing these points would strengthen the reliability of the results and help mitigate any potential biases.

Response 6:

Thank you for this thoughtful and important comment. We fully acknowledge the limitations inherent to retrospective study designs, including the risk of confounding and procedural variability. To minimize potential biases, all procedures in this study were performed using a strictly standardized workflow implemented at our institution. This workflow includes a uniform protocol for patient positioning, CT simulation, target/OAR contouring, preplanning, template fabrication, intraoperative needle guidance, and postoperative verification.

Furthermore, all procedures were conducted by a small team of senior radiation oncologists and physicists who had undergone centralized training in 3DPNCT-guided brachytherapy and had each completed over 50 implantation cases prior to the study period. This helped reduce operator variability and ensured procedural consistency across the study cohort. These details

have now been clarified in the Methods and Discussion sections to better support the internal validity of our findings**（Line 168-174,350-354）：**

To minimize procedural variability, the 3D-printed noncoplanar template-assisted CT-guided ^{125}*I seed implantation technique is performed in accordance with a standardized precision-controlled workflow, as illustrated in Figure 1. All implantations were performed by one of two senior radiation oncologists and two medical physicists with over five years of experience in brachytherapy. Each operator had completed more than 50* ^{3}*D-PNCT-guided implantation cases prior to the study period.*

Although the retrospective nature of this study introduces inherent limitations, we attempted to mitigate potential sources of bias by adhering to a highly standardized workflow and limiting procedural performance to a small group of experienced operators. This approach enhanced the internal consistency and reproducibility of the implantation procedures.

Comment 7.

While the authors state that each step of the implantation workflow is detailed, the process of designing the 3D-printed noncoplanar template remains unclear in some technical aspects: • How the STL file was created (from what input – e.g., segmentation, external surface scanning, etc.) • How the spatial registration between the CT scan (DICOM) and the 3D template (STL) was performed, • How the alignment between virtual planning and printed guide was verified before use • What software was used for merging or verifying this integration.

Response 7:

Thank you for this excellent technical question. We have now added detailed descriptions of the 3D template design process, data inputs, spatial registration methods, and both virtual and physical verification techniques used to ensure clinical accuracy. The process involved the following steps:

1. **STL File Generation**: The STL model for each 3D-printed noncoplanar template was generated using CT simulation data (DICOM format) as the primary input. Segmentation of

the skin surface, tumor target, and surrounding organs at risk (OARs) was performed using the brachytherapy treatment planning system (BTPS). Needle path coordinates, insertion angles, and depths were exported from the BTPS and imported into Mimics Innovation Suite (Materialise, Belgium) to create the STL file. No external surface scanning was required.

2. **Spatial Registration (CT to Template)**: Registration between the CT scan and the template was achieved by two methods:

For most patients, **anatomy-based registration** was used: the template's undersurface was reverse-modeled to match the 3D curvature of the patient's skin surface reconstructed from CT.

In select cases, **fiducial-based registration** was employed: small metallic markers were placed on the skin before CT scanning, and corresponding grooves were integrated into the template design to ensure unique fit.

3. **Virtual Verification**: All STL and CT-segmented models were loaded into **3D Slicer** for surface deviation analysis to ensure <2 mm deviation between the template base and patient skin.

In CAD software, needle channels were simulated to validate axial alignment with pre-planned trajectories and ensure seed positions remained within the target volume.

A virtual phantom test (digital patient model) was used to assess seed distribution, tumor coverage, and avoidance of OARs.

4. **Physical Verification**: Prior to clinical use, each template was tested on a **gelatin-based anthropomorphic phantom** embedded with fiducial markers. After mounting the template, a CT scan was acquired to compare actual needle insertion paths with planned trajectories.

Penetration accuracy was assessed by measuring the spatial deviation between planned and inserted needle tips, with a threshold of ≤2 mm for clinical acceptance.

These clarifications have been added to the Methods section under a newly defined subheading, and we appreciate the reviewer's suggestion to improve the technical completeness of the manuscript（**Line 197-213**）**:**

The STL model for each 3D-printed template was generated based on the patient's CT scan (DICOM format). Segmentation of the skin surface, target tumor, and organs at risk was conducted using the BTPS. The planned needle entry points, angles, and depths were exported from the BTPS and incorporated into the template design using Mimics (Materialise, Belgium). Spatial registration between CT and STL was maintained through two complementary strategies: (1) anatomical surface-matching, where the template undersurface was reverse-engineered to fit the patient's CT-derived body contour; and (2) fiducial-based alignment, using radiopaque markers placed on the patient during CT scanning, which were matched with physical grooves in the printed guide.

Virtual verification was performed using 3D Slicer to measure surface conformity and using CAD-based trajectory simulation to verify that needle channels aligned with planned paths. Physical accuracy was validated using a tissue-equivalent phantom with embedded fiducials, and the needle positioning deviation was measured by postoperative CT fusion. Templates were approved for use only if deviations were ≤2 mm.

Comment 8.

A reader or another institution cannot easily reproduce the procedure without: • Knowing the BTPS software used • Understanding how trajectory planning was translated to physical needle guide paths

Response 8:

We thank the reviewer for this critical and practical observation regarding procedural reproducibility. To enhance clarity and support potential replication by other institutions, we have now specified the exact brachytherapy treatment planning system (BTPS) used in this study and elaborated on the process through which virtual needle trajectories were transformed into the physical guide paths embedded within the 3D-printed template.

All preoperative planning and trajectory design were conducted using **BTPS (version 7.3，Imaging Center of the Beijing University of Aeronautics， China)**, which supports ^{125}I seed

brachytherapy with CT-based 3D modeling, target/OAR contouring, and trajectory visualization.

We have added this information to Radioactive Seed Implantation Procedure section to ensure full transparency and reproducibility of the template design and manufacturing process **（Line,184-185，208-213）:**

(BTPS，Vision7.3，Imaging Center of the Beijing University of Aeronautics and Astronautics，China)

Virtual verification was performed using 3D Slicer to measure surface conformity and using CAD-based trajectory simulation to verify that needle channels aligned with planned paths. Physical accuracy was validated using a tissue-equivalent phantom with embedded fiducials, and the needle positioning deviation was measured by postoperative CT fusion. Templates were approved for use only if deviations were ≤2 mm.

Comment 9.

Steps 6 and 7 seem to describe very similar things — both talk about using a CT scan to check if the needles are in the right place and making sure the error is less than 2 mm. It's a bit hard to tell if these are two different steps or just two ways of explaining the same thing. It might be clearer if the authors either combine them or explain more clearly how they're different.

Response 9:

We appreciate the reviewer's attention to this potential ambiguity. We agree that the distinction between Step 6 and Step 7 in our procedural description was not clearly delineated. To improve clarity:

Step 6 (Preoperative Repositioning and Calibration) refers to the initial verification step conducted **before the full implantation begins**. In this step, a **reference needle** is inserted through a designated guide path in the template, followed by a **validation CT scan**. This is used to assess whether the overall alignment between the patient, template, and planned CT space is within acceptable limits (≤2.0 mm). If not, recalibration is performed using a six-degree-of-freedom correction.

Step 7 (Verification of Needle Trajectories) is a **repetitive intraoperative quality control step** conducted during the actual insertion of multiple treatment needles. After partial or full

insertion of the needles, an additional CT scan is performed to verify each needle's position, ensuring alignment with the preplanned trajectories. Corrections are made as needed, and only once all needle paths are verified is seed implantation initiated.

We have revised the text in the Methods section to clarify this distinction and avoid potential confusion between alignment calibration and trajectory verification（**Line,214-227**）**:**

6. Preoperative Repositioning and Calibration:

Before needle insertion, the patient was immobilized, and the 3D-printed template was affixed to the skin surface. A reference needle was inserted through a predesignated trajectory. A validation CT scan (slice thickness 5 mm) was performed to assess the spatial alignment between the patient anatomy, template channels, and preoperative plan. If the deviation exceeded 2.0 mm, six-degree-of-freedom adjustments were applied for recalibration. Only when the alignment error was within acceptable limits (≤2.0 mm) did the procedure proceed.

7. Intraoperative Needle Trajectory Verification:

During the procedure, after insertion of all or part of the implantation needles, a second CT scan was obtained to verify the actual needle trajectories. The spatial coordinates of each needle were compared with the planned path. If any deviation exceeded 2.0 mm, real-time adjustments were made. This step ensured that all needle trajectories matched the preplanned paths before radioactive seed delivery.

Comment10.

"The instruments were sterilized with wet heat." The statement that instruments were sterilized using 'wet heat' is somewhat vague. It would be helpful to specify the exact sterilization method - for example, whether steam autoclaving was used, and if so, under what parameters (e.g., temperature and duration). Providing these details would improve clarity and reproducibility of the protocol.

Response 10:

Thank you for this helpful comment. We apologize for the vague phrasing. In our study, all surgical instruments—including puncture needles and implantation accessories—were disinfected using **immersion in glutaraldehyde solution at room temperature for at least 10 hours**. This method was selected to accommodate heat-sensitive components and was conducted under strict aseptic protocol according to the manufacturer's recommendations and

hospital infection control guidelines. We have revised the relevant description in the Methods section accordingly to enhance clarity and reproducibility（**Line,229-232**）：

All instruments used in the implantation procedure were disinfected by immersion in ***2% glutaraldehyde solution at room temperature for no less than 10 hours****, following standard high-level disinfection procedures for heat-sensitive medical devices.*

Comment 11.

Preoperative Treatment Planning: The BTPS was used to design the noncoplanar implantation trajectory on the basis of tumor anatomy. The seed activity (0.6–0.8 mCi) and prescribed dose (PD) were set, ensuring a D90 ≥ 95% PD for the GTV and OAR doses within acceptable limits The dose constraints for OARs are said to be "within acceptable limits" but it would be more rigorous to clearly state whether this follows an established guideline.

Response 11:

Thank you for this important comment. We agree that referring to established dose constraint guidelines improves methodological rigor and clarity. In this study, the dose constraints for organs at risk (OARs) were set based on a combination of references: the **AAPM TG-43 formalism**, previously published clinical experience in head and neck brachytherapy, and expert consensus adapted to ^{125}I permanent seed implantation. We have updated the Methods section to reflect this information and cited relevant references where appropriate（**Line,190-192**）：

Organ-at-risk (OAR) dose constraints were determined based on published head and neck brachytherapy studies and the AAPM TG-43 formalism.

Discussion

Comment 8.

The discussion includes a detailed explanation of the limitations of freehand puncture techniques in head and neck brachytherapy, which is important background. However, this information was already covered in the introduction. To avoid redundancy, the authors may consider summarizing this section more briefly or rephrasing it to focus specifically on how their findings support or address these previously mentioned challenges.

Response 8：

Thank you for this thoughtful comment. We agree that the limitations of freehand puncture were already discussed in the Introduction and that reiterating them in detail in the Discussion leads to redundancy. In response, we have revised the Discussion section to eliminate repetition and refocused this portion on how our findings specifically demonstrate the clinical utility of 3DPNCTs in addressing the limitations of freehand techniques—namely, poor needle reproducibility, angular deviation, and dose inaccuracy. This restructuring improves the focus and logical flow of the Discussion（**Line,338-345**）：

Previous studies have highlighted the technical limitations of freehand puncture in head and neck brachytherapy, including inconsistent needle angles and deviations that compromise dosimetric precision. Our study addresses these concerns by demonstrating that 3DPNCT-assisted implantation resulted in high consistency between pre- and postoperative D90, V100, and HI values. These findings confirm that template guidance effectively mitigates intraoperative variability, enhancing reproducibility and treatment accuracy in anatomically complex regions.

Comment 9.

The brief note on seed migration is appreciated, but it may be helpful to elaborate on follow-up duration and whether the study design allowed for delayed migration to be captured. A comparison with seed migration rates in the literature would also provide useful context.

Response 9：

Thank you for this valuable suggestion. In our clinical follow-up, we observed very few cases with apparent seed migration; however, systematic quantification of migration events was not included in the current study design and data collection. The follow-up period for most patients ranged from 3 to 12 months, which may allow for delayed migration to be detected radiographically if present, but we acknowledge that this aspect was not analyzed in detail.

We plan to include seed migration as a dedicated endpoint in future studies to more comprehensively evaluate its frequency, temporal characteristics, and clinical impact. A comparative analysis with reported migration rates in the literature will also be incorporated

to contextualize our findings（**Line,411-414**）**:**

Although no clinically significant seed migration was detected during routine follow-up in this cohort, systematic analysis was not performed. Future prospective tracking and correlation with published migration rates are warranted.

Comment 10.

The authors mention that integration of rapid prototyping could improve future workflows, which is promising. Expanding slightly on how automation or software advancements (e.g., intraoperative re-planning or AI-based registration) might reduce remaining sources of error would enhance the forward-looking part of the discussion.

Response 10:

We appreciate this insightful suggestion. We agree that emerging technologies such as rapid prototyping offer promising avenues to further improve the accuracy and efficiency of template-guided brachytherapy. In response, we have expanded the final paragraph of the Discussion to briefly address how these innovations may help overcome residual challenges（**Line,438-441**）**:**

Looking forward, the integration of ***rapid prototyping technologies*** *may significantly reduce the fabrication time of 3D-printed templates from several hours to under 30 minutes, enabling near real-time production and potential intraoperative use[33].*

Comment 11.

The discussion would benefit from referencing previous studies to support or contrast the current findings. Including citations of studies that have shown similar results would help validate the conclusions, while mentioning studies with different outcomes could offer a deeper understanding of the variables that influence implantation accuracy and dose distribution.

Additionally, the manuscript would be strengthened by a more forward-looking perspective. The current mention of 'rapid prototyping' is a good start, but future research directions could include exploring real-time image-guided implantation, intraoperative adaptive planning, automated needle path planning, or AI-assisted registration systems. These advancements could further minimize deviations and improve accuracy in complex anatomical regions like

the head and neck.

Response 11：

We sincerely thank the reviewer for this insightful and constructive suggestion. In response, we have revised the Discussion section to incorporate relevant studies that reported similar or contrasting results regarding implantation accuracy and dosimetric consistency in 3D-printed template-assisted brachytherapy（**Line,360-368**）**：**

Our findings of high consistency between pre- and postoperative dosimetric parameters are consistent with previous studies. Qiu et al. reported that template-assisted implantation maintained D90 and V100 deviations within 5% of preoperative plans in head and neck cancers. Similarly, Chen et al. demonstrated improved conformity indices and reduced hot spots using individualized 3D templates. In contrast, studies using freehand or standard coplanar templates often reported larger deviations and higher operator dependency. These differences highlight the role of template geometry, anatomical complexity, and procedural standardization in influencing dose accuracy.

Conclusions

Comment 1.

The conclusion restates key findings effectively, but it could be more concise by avoiding repetition of earlier points already addressed in the discussion. It would also benefit from briefly acknowledging limitations, such as the small sample size or anatomical shifts that could influence template alignment. Including these would give a more balanced and realistic summary.

Response 1：

Thank you for your thoughtful comment. We agree that the conclusion section can be improved by reducing redundancy and incorporating a brief acknowledgment of study limitations. Accordingly, we have revised the conclusion to present the core findings in a more concise form and added a sentence recognizing potential sources of uncertainty, including small sample size and anatomical shifts that may affect template alignment. This provides a more balanced and realistic summary of the study（**Line,443-450**）**：**

This study demonstrated that 3D-printed noncoplanar template-assisted CT-guided ^{125}I seed implantation achieves high dosimetric consistency between preoperative plans and

postoperative outcomes in patients with recurrent or metastatic head and neck tumors. The technique enhances implantation accuracy, reduces intraoperative variability, and supports safe dose delivery in anatomically complex regions. However, the relatively small sample size and the potential influence of anatomical shifts on template alignment highlight the need for further validation in larger, prospective studies.

Comment 2.

The authors may also consider adding a sentence about future research directions, such as integrating intraoperative imaging, real-time adaptive planning, or AI-guided needle placement to further reduce intraoperative deviations and improve outcomes.

Response 2：

Thank you for your valuable suggestion. We agree that highlighting future research directions strengthens the conclusion and connects our findings to ongoing technological advancements. We have now added a sentence outlining potential developments such as intraoperative imaging, real-time adaptive planning, and AI-assisted guidance to further enhance implantation precision（**Line,450-452**）**:**

Future research should explore the integration of intraoperative imaging, real-time adaptive planning, and AI-guided needle placement to further minimize deviations and optimize therapeutic outcomes.

Reviewer 2

Comment 1.

Considering initially "only the abstract of the article," this is important because it will either encourage or discourage a reader's intention to proceed to a full reading of the manuscript. Thus, I make the following observations regarding this section: The text used several acronyms without explaining them at their first mention; The text focused only on dosimetric precision but did not mention real clinical effectiveness objectives, such as patient survival for each dose level, limiting the clinical interpretation of the findings and potentially generating disinterest among future readers; The statistical analysis has positive points but also presents flaws, such as the lack of reporting on normality testing and a brief justification for the sample size (if space for addition or editing is possible).

Response 1:

1.**Acronym clarification:** All acronyms used in the abstract—such as GTV (gross tumor volume), D90, CI (conformity index), and HI (homogeneity index)—have now been fully spelled out upon first mention to ensure clarity for a broader readership.

2. **Clinical relevance enhancement:** While the primary objective of this study was to evaluate dosimetric consistency, we agree that contextualizing the findings in terms of their potential clinical impact improves the utility of the abstract. We have added a sentence to reflect the technique's potential to improve local control and minimize radiation exposure to adjacent organs, which are essential clinical considerations.

3. **Statistical note:** Given the space constraints of the abstract, we briefly acknowledge the use of normality testing (Shapiro-Wilk test) and the exploratory nature of the sample size. More detailed information is available in the Methods section of the full manuscript（**Line,21-39**）**:**

Objective: *Recurrent and metastatic tumors of the head and neck pose significant treatment challenges due to their proximity to critical structures and prior radiation exposure. This study aimed to evaluate the consistency between preoperative and postoperative dosimetric parameters in CT-guided ³D-printed noncoplanar template (3DPNCT)-assisted radioactive iodine-125 seed implantation (RISI).*

Methods: *Twenty-six patients with recurrent or metastatic head and neck cancer were retrospectively analyzed. Gross tumor volume (GTV) coverage and dosimetric parameters such as D90 (dose covering 90% of the GTV), conformity index (CI), and homogeneity index*

(HI) were compared before and after implantation. The Shapiro-Wilk test was used to assess data normality.

Results: *There were no significant differences between pre- and postoperative D90, V100, V150, or CI values ($P > 0.05$). Bland–Altman analysis showed high agreement for key metrics.*

Conclusions: *3DPNCT-assisted RISI demonstrated accurate dose delivery and high reproducibility. This approach may enhance local control while minimizing radiation to organs at risk in complex head and neck anatomies. These results suggest that this technique has promising clinical applicability in complex head and neck cases; however, further validation through larger prospective studies is warranted to confirm long-term efficacy and safety.*

Comment 2.

The purpose of treatment effectiveness: The study evaluated dosimetric precision (alignment between planning and execution), without evaluating clinical effectiveness (control over the progression of the malignant neoplasm, survival, and/or mortality). Thus, the title "...treatment of recurrent and metastatic tumors…" can be interpreted as referring to technical execution, but it is not possible to conclude on the real oncological benefit for the patient. No data were presented on: Tumor response rates; Disease progression; Overall survival or progression-free survival; Long-term complications.

Response 2:

Thank you for raising this important point. We fully agree that our study focused exclusively on the technical and dosimetric aspects of iodine-125 seed implantation using 3D-printed noncoplanar templates (3DPNCT), rather than evaluating clinical endpoints such as tumor response, progression, or survival outcomes. The primary objective was to assess the consistency between preoperative planning and postoperative dosimetric implementation in anatomically complex head and neck tumors.

To avoid any misinterpretation, we have the title to more accurately reflect the methodological focus and eliminate any implication of oncologic outcome evaluation. Additionally, we have clarified the scope and objective of the study in both the abstract and the introduction.

Comment 3.

Concerning the representativeness of the sample, the study involved 26 patients from a single

hospital. The sample was one of convenience and limited in size, with no sample size calculation or adequate statistical justification. In the context of the representativeness of the exposed cohort, according to the NOS, 2 out of 4 stars were awarded, that is, 50% of the possible points in this domain.

Response 3:

Thank you for this critical observation. We fully acknowledge that our study was based on a convenience sample of 26 patients treated at a single institution over a five-year period. As a retrospective feasibility analysis focused on dosimetric consistency, no a priori sample size calculation was conducted. Instead, we included all consecutive patients who met the inclusion criteria and underwent 3D-printed template-assisted ^{125}I seed implantation during the specified time frame.

While we recognize the inherent limitations of this design—particularly regarding generalizability and statistical power—the study provides an initial technical validation of the dosimetric alignment between pre- and postoperative plans. We have now explicitly stated these limitations in the revised Discussion section and clarified the nature of the sample in the Methods section. Future studies are being planned with larger cohorts and prospective sample size determination to enable more robust conclusions and potential stratified analysis **（Line,433-441）：**

The small sample size may have affected the statistical stability of the findings. A larger sample of patients are needed in future studies to validate these results. The 3D template is reconstructed from preoperative CT data, but because of delays in production, subtle anatomical changes may be observed, leading to minor mismatches between the template and anatomical structures intraoperatively. Looking forward, the integration of rapid prototyping technologies may significantly reduce the fabrication time of 3D-printed templates from several hours to under 30 minutes, enabling near real-time production and potential intraoperative use[33].

Comment 4.

Thank you for your detailed and methodical assessment based on the Newcastle–Ottawa Scale (NOS). We fully acknowledge that the retrospective design and single-center origin of our cohort limit the external generalizability of our findings. The sample size was relatively small (n = 26), and no a priori power analysis was conducted. However, we would like to clarify the

following to improve transparency regarding the sample selection process and its representativeness:

Response 4:

1.**Consecutive inclusion:** All patients who met the predefined inclusion criteria and underwent 3D-printed noncoplanar template-assisted ^{125}I seed implantation at our institution between January 2018 and December 2022 were included consecutively, without selection filtering. This minimizes intentional selection bias.

2.**Clinical diversity within the cohort:** Although the sample was modest in size, it included a heterogeneous set of head and neck cancer cases—nasopharyngeal, laryngeal, thyroid, hypopharyngeal, buccal mucosa, maxillary sinus, parotid gland, and floor of mouth tumors—thus reflecting real-world anatomical and clinical diversity often encountered in salvage brachytherapy.

3.**Objective of the study:** The primary aim of the study was to evaluate **technical dosimetric consistency**, not treatment efficacy. The observational, exploratory nature of the study justified the inclusion of all available eligible patients over the defined period, consistent with early-stage feasibility research.

We have added this clarification to both the Methods and Discussion sections to better convey the rationale and limitations of our sample framework. Future studies will aim to validate these findings in larger, multi-center cohorts with formal sample size estimation **（Line,433-431）：**

The small sample size may have affected the statistical stability of the findings. A larger sample of patients are needed in future studies to validate these results. The 3D template is reconstructed from preoperative CT data, but because of delays in production, subtle anatomical changes may be observed, leading to minor mismatches between the template and anatomical structures intraoperatively. Looking forward, the integration of rapid prototyping technologies may significantly reduce the fabrication time of 3D-printed templates from several hours to under 30 minutes, enabling near real-time production and potential intraoperative use.

Comment 5.

Regarding the control of confounding factors, no formal control was observed, as there was no comparative group or multivariate analysis. Thus, in the context of comparability, according

to the principles of the NOS, no stars were awarded, resulting in no score in this quality domain.

Response 5:

We thank the reviewer for this important observation. We fully acknowledge that our study design lacks a control group and does not incorporate statistical adjustments such as stratification or multivariate analysis to account for potential confounding factors. This limitation is inherent to the retrospective single-arm nature of our investigation, which was not intended to compare different techniques but rather to assess the internal consistency between planned and delivered dosimetric parameters in 3DPNCT-assisted seed implantation.

Given the study's methodological focus on **technical accuracy and reproducibility**, we did not aim to evaluate comparative efficacy (e.g., template-guided vs. freehand implantation) or control for confounders related to tumor location, stage, or patient characteristics. However, we agree that these are important areas for future inquiry. We have now clearly acknowledged this limitation in the revised Discussion section and are planning prospective comparative studies that include both template-guided and freehand arms, as well as stratified subgroup analysis based on anatomical and clinical variables（**Line,421-432**）**:**

An important limitation of this study is the inclusion of patients with diverse tumor types and anatomical locations. Variations in tumor depth, local tissue compliance, and adjacency to organs at risk may influence needle placement accuracy and, consequently, dosimetric consistency. While this heterogeneity mirrors routine clinical practice, it may obscure location-specific performance differences of the 3D-printed template system. Future studies with larger cohorts should consider stratified or subgroup analyses to evaluate anatomical factors affecting implantation precision.

Comment 6.

Regarding the outcome not being present at the start of the study, it was appropriate for dosimetry purposes; however, the clinical outcome of oncological effectiveness, such as survival or mortality rates, unfortunately, was not the focus. Regarding the follow-up time, it was not subject to evaluation, as the focus was only on immediate dosimetry, unfortunately not addressing long-term clinical outcomes.

Response 6:

We appreciate the reviewer's careful and fair evaluation of the outcome domain. As correctly noted, our study focused exclusively on the technical dosimetric consistency between

preoperative and postoperative plans, and did not include long-term clinical outcomes such as tumor response, progression-free survival (PFS), overall survival (OS), or treatment-related toxicities.

This was a deliberate design choice, as the primary objective was to assess the feasibility and spatial precision of 3DPNCT-assisted ^{125}I seed implantation in anatomically complex head and neck tumors. While we agree that oncologic outcomes are critical to understanding the full therapeutic value of this technique, they were beyond the scope of this retrospective dosimetric validation.

Nonetheless, we recognize the importance of bridging the gap between technical accuracy and real-world clinical benefit. As part of our future research agenda, we are initiating a prospective clinical study that will incorporate long-term follow-up to assess tumor response, local control rates, progression-free survival, and late complications（**Line,428-432**）**:**

While this study confirmed the dosimetric precision of 3DPNCT-assisted implantation, it did not evaluate clinical endpoints such as tumor response or patient survival. Future prospective studies with structured follow-up are necessary to correlate dosimetric parameters with long-term oncologic outcomes and treatment-related toxicity profiles.

Comment 7.

Confounding bias: Heterogeneity of tumors, involving different types, stages, and anatomical locations, without statistical adjustment.

Response 7:

Thank you for this thoughtful and accurate observation. We agree that the inclusion of diverse tumor types and anatomical subsites in the head and neck region introduces clinical and geometrical heterogeneity, which may affect the accuracy of needle placement, dose distribution, and overall dosimetric consistency.

In this retrospective study, we did not perform stratified subgroup analysis or statistical adjustments for tumor type, depth, or anatomical location, primarily due to the limited sample size. Our goal was to provide a preliminary technical validation of template-guided dose reproducibility across a real-world heterogeneous cohort, rather than draw subtype-specific conclusions.

We have now explicitly acknowledged this limitation in the Discussion section and are designing follow-up studies with larger cohorts that will include subgroup comparisons (e.g.,

deep vs superficial tumors, anterior vs posterior sites, radiosensitive vs radioresistant histologies). This will allow more refined analyses of how anatomical and pathological variables influence implantation accuracy and clinical outcomes **（Line,421-432）：**

An important limitation of this study is the inclusion of patients with diverse tumor types and anatomical locations. Variations in tumor depth, local tissue compliance, and adjacency to organs at risk may influence needle placement accuracy and, consequently, dosimetric consistency. While this heterogeneity mirrors routine clinical practice, it may obscure location-specific performance differences of the 3D-printed template system. Future studies with larger cohorts should consider stratified or subgroup analyses to evaluate anatomical factors affecting implantation precision.

Comment 8.

The sample size calculation was not performed, and no justification was provided. Normality testing was not mentioned, yet the paired t-test was used, which presumes prior testing. Also, the study did not explore whether different dose levels (90–120 Gy) affected dosimetric coverage such as V100, which could have been investigated using ANOVA or Kruskal-Wallis. These statistical omissions, along with methodological limitations, prevent generalizing the findings to clinical treatment effectiveness.

Response 8:

We thank the reviewer for this comprehensive and insightful evaluation of the statistical methodology.

1.Sample Size Justification:

We agree that no a priori sample size calculation was conducted, as the study was designed as a retrospective feasibility assessment. All eligible patients within the study window were included consecutively. We have clarified this in both the Methods and Discussion sections. **（Line,138-142）：**

This retrospective study included all patients who met the eligibility criteria within the five-year recruitment window. No a priori power calculation was performed, as the study was exploratory in nature and focused on assessing dosimetric reproducibility rather than hypothesis-driven clinical outcome comparison.

2.**Normality Testing and Test Selection:**

We acknowledge the importance of testing data normality prior to choosing statistical methods. In the revised manuscript, we now explicitly state that the **Shapiro–Wilk test** was performed for all dosimetric variables. Where the data were normally distributed, **paired t-tests** were used; where not, **Wilcoxon signed-rank tests** were applied. These updates are reflected in the Statistical Analysis subsection. **（Line,287-290）：**

The Shapiro–Wilk test was applied to assess the normality of the distribution of each dosimetric parameter. For normally distributed variables, the paired t-test was used; for non-normally distributed data, the Wilcoxon signed-rank test was used.

3.**Subgroup Analysis by Dose Level (90–120 Gy):**

We appreciate the reviewer's suggestion regarding dose stratification. Indeed, we did not perform subgroup comparisons across different prescribed dose levels due to limited statistical power in the current sample. However, we agree that this is a valuable avenue for future research. In response, we have added a note in the Discussion acknowledging this missed opportunity and our plan to explore this in larger future cohorts using ANOVA or Kruskal–Wallis tests, depending on the distributional assumptions. **（Line,415-420）：**

Although the prescribed doses ranged from 90 to 120 Gy, no subgroup comparison was performed to assess whether higher doses improved GTV coverage (e.g., V100). This was due to limited statistical power in the current sample. In future studies with larger cohorts, we plan to stratify patients by prescription dose and use ANOVA or Kruskal–Wallis testing to evaluate dose-response effects.

4.**Generalizability and Clinical Interpretation:**

We respectfully agree that the current study, focusing solely on dosimetric reproducibility, cannot provide evidence of oncologic treatment effectiveness. We have revised the title, abstract, and conclusion to clarify the study's technical scope and have more clearly acknowledged in the Discussion that generalizability is limited by the lack of clinical outcome assessment and subgroup control. **（Line,131-132）：**

Therapeutic effectiveness was not evaluated in this study.

Reviewer 3

Comment 1.

Well thought out. Appropriate statistics. Good explanation of methodology. Minimal discussion on limitations, however may be appropriate for this type of proof of concept and will need further assessment.

Response 1:

We sincerely thank the reviewer for the encouraging comments and constructive feedback. We are pleased that the study design, statistical methodology, and technical rationale were found to be well structured and appropriate. As this was intended as a technical proof-of-concept study, we acknowledge the limitations in clinical scope and generalizability. In response, we have strengthened the Discussion section to more clearly articulate these limitations and outlined future directions for clinical validation and stratified analysis. **（Line,421-432）：**

An important limitation of this study is the inclusion of patients with diverse tumor types and anatomical locations. Variations in tumor depth, local tissue compliance, and adjacency to organs at risk may influence needle placement accuracy and, consequently, dosimetric consistency. While this heterogeneity mirrors routine clinical practice, it may obscure location-specific performance differences of the 3D-printed template system. Future studies with larger cohorts should consider stratified or subgroup analyses to evaluate anatomical factors affecting implantation precision. While this study confirmed the dosimetric precision of 3DPNCT-assisted implantation, it did not evaluate clinical endpoints such as tumor response or patient survival. Future prospective studies with structured follow-up are necessary to correlate dosimetric parameters with long-term oncologic outcomes and treatment-related toxicity profiles.

Reviewer 4

Comment 1.

More number of patients and combined studies should be considered in future in such a study. Such studies can help in further making brachytherapy more precise as we tend to eliminate human error of free puncture.

Response 1:

We sincerely thank the reviewer for the encouraging remarks and future-oriented suggestions. We fully agree that expanding the patient cohort and conducting multicenter or combined studies will be essential to validate the broader applicability of 3DPNCT-guided brachytherapy. As noted, template assistance has the potential to significantly reduce operator-dependent variability and improve implantation precision compared to freehand techniques. We have now highlighted these future directions in the revised Discussion section and are actively planning follow-up studies with larger sample sizes and comparative designs. **（Line,411-420）：**

Although no clinically significant seed migration was detected during routine follow-up in this cohort, systematic analysis was not performed. Future prospective tracking and correlation with published migration rates are warranted.

Although the prescribed doses ranged from 90 to 120 Gy, no subgroup comparison was performed to assess whether higher doses improved GTV coverage (e.g., V100). This was due to limited statistical power in the current sample. In future studies with larger cohorts, we plan to stratify patients by prescription dose and use ANOVA or Kruskal–Wallis testing to evaluate dose-response effects.
