# Supplementary material for: Dosimetry study of 3D-printed noncoplanar template-assisted CT-guided 125I seed implantation for the treatment of recurrent and metastatic tumors in the head and neck
Source: PLoS One. 2025 Aug 14;20(8):e0330271. doi: 10.1371/journal.pone.0330271 (PMC12352675; doi:10.1371/journal.pone.0330271)
Supplement: Supplementary file 4 — Responses_to_reviewers_PLOSONE.docx [file pone.0330271.s004.docx]

**Responses_to_reviewers_PLOSONE**

**Reviewer 2**

**Discussion**

**Comment 1.**

Small and heterogeneous sample: A sample of 26 patients with multiple tumor types and anatomical sites, making generalizations difficult. (It is recommended that this be noted in the discussion, before the final conclusion, as a limitation of the study).

**Response 1：**

Thank you for this thoughtful comment. We have indicated in the discussion section that this study has a small sample size and involves diverse tumor types and anatomical locations.**（Line,433-435）:**

*The small sample size may have affected the statistical stability of the findings. A larger sample of patients are needed in future studies to validate these results.*

**Comment 2.**

Lack of stratified analysis.

**Response 2：**

Thank you for this valuable suggestion. Due to the limited sample size, stratified analyses were not performed, and this has been explicitly stated.**（Line,442-443）:**

*Stratified analyses were not performed due to limited sample size, which may mask subgroup-specific dosimetric differences.*

**Comment 3.**

Absence of clinical outcomes – Local control, survival, or toxicity were not assessed.

**Response 3：**

Thank you for this valuable suggestion. We have clearly stated that clinical outcomes were not evaluated in this study, highlighting the need for prospective follow-up studies in the future.**（Line,428-432）:**

*While this study confirmed the dosimetric precision of 3DPNCT-assisted implantation, it did not evaluate clinical endpoints such as tumor response or patient survival. Future prospective studies with structured follow-up are necessary to correlate dosimetric parameters with long-term oncologic outcomes and treatment-related toxicity profiles.*

**Comment 4.**

Lack of comparison with other techniques – No comparison with manual technique, coplanar guides, or robotic approaches.

**Response 4：**

Thank you for this valuable suggestion. The manuscript already mentions the limitation of lacking comparisons with other techniques, such as manual methods or alternative template-based approaches.**（Line,365-366）:**

*In contrast, studies using freehand or standard coplanar templates often reported larger deviations and higher operator dependency[32].*

**Comment 5.**

Limited safety assessment – Only immediate evaluation of seed migration, without follow-up.

**Response 5：**

Thank you for this valuable suggestion. We have emphasized that safety assessment was restricted to immediate evaluation of seed migration, and additional relevant content has been included accordingly.**（Line,443-444）:**

*Long-term safety assessments were not conducted, limiting conclusions about persistent seed migration risks.*

**Comment 6.**

Selection bias: Retrospective, convenience sampling (It is recommended that this be noted in the discussion, before the final conclusion, as a limitation of the study).

**Response 6：**

Thank you for this valuable suggestion. We have highlighted the issue of selection bias in the discussion, with corresponding content added.**（Line,446-447）:**

*The retrospective design and convenience sampling introduce potential selection bias*

**Comment 7.**

Measurement bias – Image fusion subject to errors (It is recommended that this be noted in the discussion, before the final conclusion, as a limitation of the study).

**Response 7：**

Thank you for this valuable suggestion. We explicitly indicated the potential measurement bias resulting from errors in image fusion.**（Line,444-446）:**

*Measurement bias from potential image fusion errors between pre- and postoperative scans could affect dosimetric accuracy.*

**Comment 8.**

Operator bias – Performed by a highly specialized team, limiting generalization (should be highlighted to the clinical community).

**Response 8：**

Thank you for this valuable suggestion. We have underscored in the discussion that operator expertise may limit the generalizability of the study, with appropriate content added.**（Line,350-354）:**

*Although the retrospective nature of this study introduces inherent limitations, we attempted to mitigate potential sources of bias by adhering to a highly standardized workflow and limiting procedural performance to a small group of experienced operators. This approach enhanced the internal consistency and reproducibility of the implantation procedures.*

**Comment 9.**

Possibly Overestimated Results.

**Response 9：**

Thank you for this valuable suggestion. We have emphasized the need for cautious interpretation of the results.**（Line,447-449）:**

*Caution is necessary in interpreting the clinical applicability of these promising results, given the current absence of conclusive clinical outcomes.*

**Comment 10.**

Partial dosimetric consistency: D100 and CI showed significant differences.

**Response 10：**

Thank you for this valuable suggestion. We have explicitly noted significant differences in the D100 and CI parameters in both the results and discussion sections, along with potential explanations.**（Line,379-394）:**

*However, this study revealed that the pre- and postoperative D100 and CI values were significantly different (P < 0.05), suggesting that the postoperative dose distribution may still deviate to some extent from the preoperative distribution. Possible explanations include the following:*

*Minor errors in patient positioning: Although vacuum cushions and other fixation devices were used intraoperatively to increase positioning reproducibility, slight positioning errors may still have occurred, leading to subtle variations in needle direction and depth. Needle deviation due to tissue resistance: During needle insertion, the implantation needle must pass through various tissues and bony structures, where nonuniform resistance may cause slight needle deformation, leading to positional deviations. Image registration errors between preoperative and postoperative scans: The time interval between preoperative and postoperative CT scans may result in slight tissue shifts, affecting the precise alignment of the target volume and thereby introducing minor variations in dose distribution.*

**Comment 11.**

Optimistic clinical applicability: Are there conclusive clinical data?

**Response 11：**

Thank you for this valuable suggestion. We have highlighted that further clinical data is necessary before widespread clinical adoption.**（Line,449-451）:**

*Despite promising dosimetric results, definitive clinical applicability requires conclusive clinical data from future prospective studies.*

**Comment 12.**

lity not proven – No follow-up for late migration (It is recommended that this be noted in the discussion, before the final conclusion, as a limitation of the study).

**Response 12：**

Thank you for this valuable suggestion. We have clearly stated that long-term follow-up to assess seed stability was not conducted.**（Line,406-414）:**

*In clinical practice, seed migration or loss may occur after implantation. In this study, immediate postoperative CT revealed no seed displacement. However, tissue interactions and patient movement may still contribute to postoperative seed migration. To reduce this risk, seeds can be implanted in a chain link configuration. Additionally, regular CT follow-ups are recommended to monitor seed stability. Although no clinically significant seed migration was detected during routine follow-up in this cohort, systematic analysis was not performed. Future prospective tracking and correlation with published migration rates are warranted.*
